# Cyclin-dependent Kinase 5: Novel role of gene variants identified in ADHD

**DOI:** 10.1038/s41598-017-06852-2

**Published:** 2017-07-28

**Authors:** Subhamita Maitra, Mahasweta Chatterjee, Swagata Sinha, Kanchan Mukhopadhyay

**Affiliations:** Manovikas Biomedical Research and Diagnostic Centre, E.M. Bypass, Kolkata, India

## Abstract

Cortical neuronal migration and formation of filamentous actin cytoskeleton, needed for development, normal cell growth and differentiation, are regulated by the cyclin-dependent kinase 5 (Cdk5). Attention deficit hyperactivity disorder (ADHD) is associated with delayed maturation of the brain and hence we hypothesized that cdk5 may have a role in ADHD. Eight functional CDK5 gene variants were analyzed in 848 Indo-Caucasoid individuals including 217 families with ADHD probands and 250 healthy volunteers. Only three variants, rs2069454, rs2069456 and rs2069459, predicted to affect transcription, were found to be bimorphic. Significant difference in rs2069456 “AC” genotype frequency was noticed in the probands, more specifically in the males. Family based analysis revealed over transmission of rs2069454 “C” and rs2069456 “A” to the probands. Quantitative trait analysis exhibited association of haplotypes with inattention, domain specific impulsivity, and behavioral problem, though no significant contribution was noticed on the age of onset of ADHD. Gene variants also showed significant association with cognitive function and co-morbidity. Probands having rs2069459 “TT” showed betterment during follow up. It may be inferred from this pilot study that CDK5 may affect ADHD etiology, possibly by attenuating synaptic neurotransmission and could be a useful target for therapeutic intervention.

## Introduction

Attention deficit hyperactivity disorder (ADHD) is a developmental disorder often characterized by dysfunction of the synaptic system^1^. Major symptoms of the disorder include age inappropriate inattention, hyperactivity and/or impulsivity^2^. Though mostly detected in school-going children, ADHD is also diagnosed in adults^3^. Cognitive problems, as a result of poor information processing, have also been reported in ADHD patients^4^. Further, a number of psychiatric disorders co-occur with ADHD^2^. Among them, oppositional defiant disorder (ODD) and learning difficulties (LD) are the most common co-morbid conditions amongst Indian^5^ as well as Caucasoid^2^ populations. Long term follow up of ADHD probands revealed personality disorder, substance abuse and/or criminal behaviors in adults^6^, indicating persistence of traits. Thus, early recognition of the condition becomes necessary for proper intervention^7^.

Whether ADHD is caused by delayed or atypical maturation of the brain, as compared to the normal brain, remained a matter of conjecture till date^8^. During resting phase or excited stage, ADHD children showed brain activity similar to younger normal children^9, 10^. Specific maturational delay in connection between the default mode network and task positive networks was also reported^11^. Abnormal circuitry propagation or atypical development was also evidenced^12–14^. Imaging study documented slower cortical development as a strong basis of ADHD^15^. Pattern of development of the cortical thickness was comparable in both ADHD and healthy children, but a significant difference in the initiation time^15^, with almost a 2–5 years delay in the cortical points (LPFC, temporal, occipital cortex), was noticed as compared to normal healthy brain. However, development in primary motor cortex peak thickness was not that delayed^15^. Diffusion tensor imaging revealed decreased neural branching in children with ADHD^16^. A difference in the developmental trajectory of the caudate was also observed^17^. A meta-analytic study showed hypo-activation of the fronto-parietal network regulating attention, while somato-motor network exhibited hyper-activation^18^. Thus, the balance between cortical thickening during early childhood and cortical thinning during pubertal stage may be disrupted in ADHD patients^15^. This may eventually lead to an altered over all brain activity and expression of ADHD associated symptoms. Siblings of individuals with ADHD also exhibited disruption in white matter, thereby indicating a strong familial basis^19^.

The Cyclin-dependent kinase 5 (Cdk5) is a proline-directed serine/threonine kinase crucial for normal development^20^, since absence of CDK5 leads to lethality^21^. Two homologous non-cyclin activators, p35 (CDK5R1) and p39 (CDK5R2), aid in the function of Cdk5 and their differential expressions in mice during development indicate individualized roles in the adult brain too^22^. Phosphorylation of double cornitine (DCX), crucial for neuronal migration in the cortex, is mediated by Cdk5. A mutation at the 297^th^ serine residue of DCX led to loss of function of the protein as compared to pharmacological inhibition of Cdk5^23^. Cdk5 mediated phosphorylation of Focal Adhesion Kinase (FAK) is also crucial for neuronal migration through regulation of FORK, a microtubule important for nuclear translocation^24^. Further, Cdk5 induced activation of Wiskott-Aldrich syndrome family member 1 proteins in the neurons was reported to play major roles in the formation of filamentous actin cytoskeleton and therefore, needed for development as well as normal cell growth and differentiation^25^. Maintenance of neuron-astrocyte integrity in the developing brain through connexin was also reported to be regulated by Cdk5^26^.

CDK5R1 knockout mice (with nonfunctional Cdk5) were reported to exhibit hyperactivity^27^, a major trait observed in ADHD probands. Neuroanatomical analysis revealed delayed maturation in the cortical regions of the brain of ADHD probands^15^. Gene expression database (BGEE) exhibited Cdk5expression in several cortical regions including the frontal cortex, dorsolateral prefrontal cortex and caudate nucleus during the developmental stages (ST1). On the contrary, the primary motor cortex, which showed early maturation in ADHD probands as compared to typically developing children^15^, was devoid of Cdk5 expression during infancy, pubertal or adolescent stages (BGEE analysis). On the basis of various neurobiological functions and localization of Cdk5, we hypothesized that aberrant Cdk5 expression during the developmental period may interfere with cortical maturation observed in ADHD probands, thus affecting the etiology of ADHD. Since one of the easiest ways to identify gene function is by looking into functional genetic polymorphisms, we analyzed genomic DNA samples of 848 individuals, including unrelated nuclear families with ADHD probands and ethnically matched controls, to identify contribution of *CDK5* gene variants in the etiology of ADHD. Association of different gene variants with endophenotypes of the probands including, sex, age of onset, inattention, hyperactivity, impulsivity, behavioral problem, co-morbid disorders and executive deficit, were also analyzed in order to find out contribution of gene variants on these traits. Finally probands were re-assessed to find out association of the variants with persistence of the disease severity.

## Results

Three genomic regions of CDK5 were analyzed by DNA sequencing. Exon 2, containing the binding site, and its flanking regions were analyzed in 100 samples and no bimorhic site was detected in a stretch of 801 bp. Hence, this site was not analyzed any further. Two other sites analyzed were a stretch of 868 bp, including the exon 6 containing the active site and the exon 10 with its flanking regions (780 bp) covering rs2069459 already studied in relation to different diseases including malignancy. These last two stretches were analyzed in all the samples by DNA sequencing to detect frequencies of eight reported SNPs (NCBI).

Out of eight SNPs, only three, rs2069454 (M1), rs2069456 (M2) and rs2069459 (M3), were found to be bimorphic in the studied population. *In silico* analysis showed that all three may affect transcription (ST2). Other five variants, rs2069453, rs1057766, rs2069460, rs11541602 and rs2069455, though may change Cdk5 activity, were found to be monomorphic in this population (ST2). Statistical analysis was performed for these three bimorphic variants only. The data obtained after 1000 permutation were presented and thus hold true for multiple correction.

### Case-control analysis

Control samples deviated marginally (0.02) from the Hardy-Weinberg equilibrium (HWE) for M2. Further analysis revealed that only the male control individuals deviated from the HWE. All other sites followed the HWE (P > 0.05).

No statistically significant difference was observed for M1 (Table 1). However, stratified analysis on different subtypes revealed higher frequency of the “G” allele and “GG” genotypes (Power > 80%) in subjects belonging to the hyperactive (HAS) and combined (CS) subtypes (ST3; P < 0.02).

**Table 1:** Allele and genotype frequency of CDK5 bimorphic variants in the Indo-Caucasoid population.

Variant	Allele/Genotype	Control	Probands		Father		Mother	
		Frequency	Frequency	χ2(P)*	Frequency	χ2(P)*	Frequency	χ2(P)*
rs2069454 (M1)	G	0.93	0.94	0.22 (0.63)	0.94	0.22 (0.64)	0.94	0.12 (0.73)
	C	0.07	0.06		0.06		0.06	
	GG	0.87	0.89	2.10 (0.35)	0.89	0.23 (0.63)	0.88	0.13 (0.72)
	GC	0.13	0.107		0.11		0.12	
	CC	0.0	0.005		0.0		0.0	
rs2069456 (M2)	A	0.76	0.78	0.88 (0.35)	0.73	0.67 (0.41)	0.70	2.11 (0.14)
	C	0.24	0.22		0.27		0.30	
	AA	0.60	0.60	5.12 (0.077)	0.48	15.61 (0.0004)	0.47	9.69 (0.007)
	AC	0.31	0.36		0.50		0.47	
	CC	0.09	0.04		0.02		0.06	
rs2069459 (M3)	G	0.61	0.64	0.45 (0.50)	0.64	0.36 (0.55)	0.64	0.34 (0.56)
	T	0.39	0.36		0.36		0.36	
	GG	0.40	0.41	0.43 (0.81)	0.40	1.03 (0.60)	0.41	0.75 (0.69)
	GT	0.43	0.45		0.47		0.46	
	TT	0.17	0.14		0.13		0.13	

NB.* as compared to control.

M2 revealed a bias in occurrence of the “AC” genotype in the probands (P = 0.07; OR for “A” 1.12, CI 0.58 to 2.16; Power 51%, Table 1). Stratified analysis showed significant association for the male probands as compared to gender matched controls (χ^2^ = 7.33, P = 0.025, OR for “A” 1.18, CI 0.61 to 2.27; Power 68%). “AC” genotype frequency was higher in the parents of probands also (P < 0.01; Power = 95%, Table 1) with concomitantly lower occurrence of the “AA” genotype. Comparative analysis on AC/AA revealed higher OR for the father (OR = 0.50, CI = 0.28–0.89) as well as the mother (OR = 0.52, CI = 0.29–0.93) with respect to the control, while no significant data was obtained for the proband. Frequency of this genotype was also higher in the inattentive (IAS) and CS subtypes while the HAS showed higher frequency of “AA” making a statistically significant difference (ST3; χ^2^ = 13.1, P < 0.01; Power > 80%).

M3 failed to show any significant difference (Table 1).

### Comparative analysis on haplotype frequency

A number of haplotypes showed significant difference in occurrence (ST4). M1-M2 “C-C” haplotype was absent in the probands and stratified analysis showed a marginally significant difference for the male individuals (χ^2^ = 4.37, P = 0.036) as opposed to male controls. The “C-T” haplotype formed by M2-M3 also showed a trend for association (χ^2^ = 2.80, P = 0.09). M1-M3 “C-G” haplotype revealed statistically significant association (χ^2^ = 4.85, P = 0.03), which was principally due to significantly lower occurrence in the male probands (χ^2^ = 4.34, P = 0.037). The C-C-G haplotype formed by the three markers showed lower occurrence in the probands (χ^2^ = 3.48, P = 0.06), which was statistically significant for the male probands (χ^2^ = 32.73, P = 1.05e–008). However, power of all these analyses was <50% thus indicating necessity for further validation. The parental population failed to show any significant difference in the occurrence of haplotypes (data not presented for brevity).

### Linkage Disequilibrium (LD) analysis

**Figure 1:**
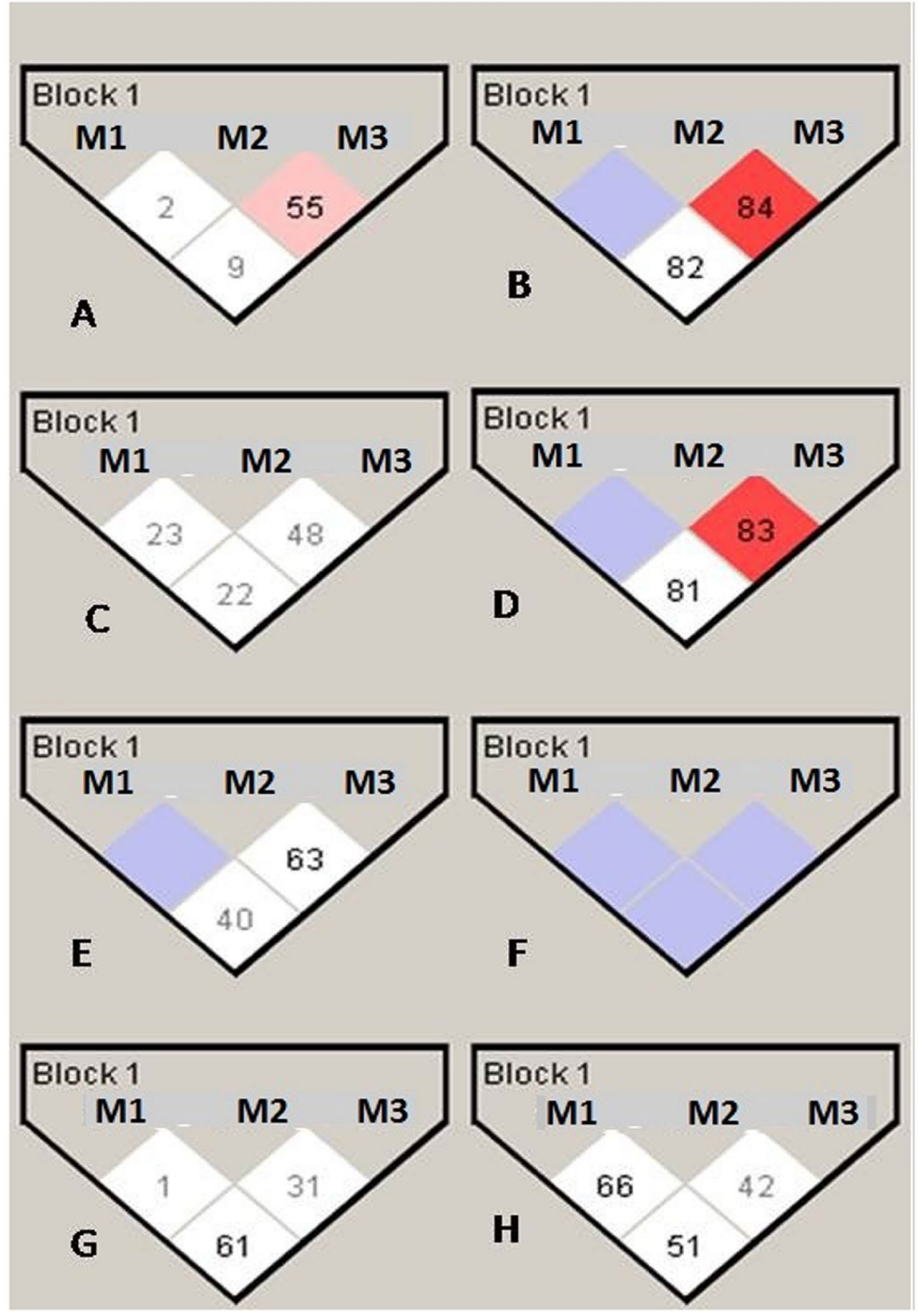
LD between CDK5 variants in all controls (**A**), all probands (**B**), male controls (**C**), Male probands (**D**), female controls (**E**), female probands (**F**), Father of probands(**G**), Mother of probands (**H**). Variants (M1,2,3); rs2069454, rs22069456, rs2069459.

The three SNPs residing at a distance of 373 basses (M1-M2) and 1686 bases (M2-M3) revealed lack of LD in the control as well as parental populations (Fig. 1). On the other hand, in the ADHD probands, more so for the male probands, strong LD was observed between M2 and M3 (LOD > 4.0, D’ > 0.80, R^2^ > 0.10). The female probands also showed positive LD between these two sites; however, the LOD score was less indicating absence of true LD.

### Parental transmission analysis

Marginal over transmission of M2 “A” was noticed from the parents (Table 2; χ^2^ = 4.05, P = 0.04, OR 1.89, CI 0.89 to 3.40, Power 52%), which was basically maternal in nature (χ^2^ = 3.70, P = 0.05, OR 2.19, CI 1.02 to 4.69, Power 54%). No significant haplotypic transmission bias was found from either parent excepting for a preferential maternal transmission of G-A and C-A of M1 and M2 (χ^2^ = 6.46, P = 0.04, Power 55%) (ST5). Haplotypes with M3 “T” also showed a trend for higher transmission {χ^2^ = 5.01, P = 0.08, Power 47% (ST5)}.

**Table 2:** Familial allelic transmission analyzed by Haplotype based haplotype relative risk test.

Variant	Allele	Parental			Paternal			Maternal			Maternal age < 30 yrs*		
		T	NT	LRS (P)	T	NT	LRS (P)	T	NT	LRS (P)	T	NT	LRS (P)
M1	G	0.95	0.97	1.20 (0.27)	0.94	0.97	0.53 (0.47)	0.96	0.98	0.70 (0.40)	0.94	1.0	4.26 (0.04)
	C	0.05	0.03		0.06	0.03		0.04	0.02		0.06	0.0	
M2	A	0.87	0.78	4.05 (0.04)	0.86	0.80	0.81 (0.37)	0.88	0.77	3.70 (0.05)	0.88	0.76	2.06 (0.15)
	C	0.13	0.22		0.14	0.20		0.12	0.23		0.12	0.24	
M3	G	0.64	0.69	1.01 (0.32)	0.64	0.70	0.77 (0.38)	0.64	0.68	0.31 (0.58)	0.59	0.71	1.84 (0.18)
	T	0.36	0.31		0.36	0.30		0.36	0.32		0.41	0.29	

Stratified analysis based on maternal age during the birth of the proband (Table 2) revealed significant over transmission of M1 “C” allele (χ^2^ = 4.26, P = 0.04, OR 0.13, CI 0.03 to 0.65, Power 54%) from mothers below 30 years of age (Mean age 24.12 ± 5.26) while no bias was found for mothers on or above 30 years of age (Mean age 33.15 ± 5.32).

### Relation between markers, age of onset and disease severity

None of the markers showed any significant association with the age of onset of the disorder as well as disease severity measured by CPRS-R (data not presented).

### Association of markers with endophenotypes

#### Inattention (IA)

Quantitative trait analysis (QTA) revealed that M2 “C” allele had positive effect on DSM-IV-TR IA score (Add value = 0.08, CI 0.02 to 0.14, χ^2^ = 6.58, P = 0.01), while no association was found for the same trait measured through CPRS-R. M2 “CC” genotype also exhibited a trend of association with higher IA score (Add value = 0.20, CI 0.01 to 0.40, χ^2^ = 3.06, P = 0.08). The same allele as a part of the G-C haplotype, formed by M1-M2, also exhibited a positive effect on IA (Table 3; P = 0.004). Other variants failed to show any association with this trait.

**Table 3:** Quantitative Trait analysis involving ADHD associated phenotypes and gene variants.

Phenotype	SNP combination	Haplotypes	Add value	CI	χ2(P)
IA-DSM	M1-M2	G-C	0.09	0.02 to 0.15	7.93 (0.004)
HA-DSM	M2-M3	C-T	−0.27	−0.64 to 0.10	2.93 (0.09)
	M1-M2-M3	G-C-T	−0.28	−00.63 to 0.06	3.45 (0.06)
IMP-DSM	M1-M2	C-A	−0.33	−0.40 to 0.0	4.55(0.03)
	M2-M3	C-T	−0.92	−2.05 to 0.19	5.97 (0.01)
	M1-M2- M3	C-A-T	−0.44	−0.85 to −0.01	3.92 (0.05)
IMP-Int-Personal	M2-M3	A-T	−0.09	−0.17 to −0.01	5.14 (0.02)
	M1- M3	G-T	−0.08	−0.15 to −0.003	4.61 (0.03)
	M1-M2- M3	G-A-T	−0.09	−0.18 to −0.01	5.12 (0.02)
CPRS-Bpr	M1-M2	C-A	−0.10	−0.19 to −0.005	5.44 (0.02)
	M2-M3	A-T	−0.06	−0.11 to −0.01	6.92 (0.008)
	M1-M3	G-T	−0.05	−0.10 to −0.01	4.61 (0.03)
	M1-M2-M3	G-A-T	−0.06	−0.11 to −0.002	5.16 (0.02)

N.B: IA-DSM-Inattention score DSM IV; HA-DSM-hyperactivity score DSM-IV; IMP-DSM-impulsivity score DSM; Int-Per-IMP- TSI- interpersonal impulsivity score Tsukayama scale for impulsivity; CPRS-Bpr-Conner’s parent rating scale behavioral problem score.

#### Hyperactivity (HA)

No significant association of any allele or genotype was noticed for this endophenotype. Haplotype analysis revealed negative effect of two haplotypes, M2-M3 “C-T” and M1-M2-M3 “G-C-T” on the DSM-IV-TR HA score (Table 3; P < 0.09).

#### Impulsivity (Imp)

Average impulsivity (Avg-Imp) score was negatively affected by the M2 “CC” genotype (Add value = −0.60, CI −1.21 to 0.008, χ^2^ = 4.62, P = 0.03) while no such effect was observed for the “C” allele. A trend for negative impact of M3 “T” (Add value = − 0.06, CI −0.12 to 0.004, χ^2^ = 3.30, P = 0.07) was also noticed on Inter-Personal Impulsivity (Int-Per-Imp) measured through Tsukayama Scale of Impulsivity (TSI). Haplotype analysis demonstrated negative impact of M1-M2 “C-A” and M1-M2-M3 “C-A-T” on DSM-IV-TR Imp (Table 3; P < 0.05). Negative effects of three haplotypes were also observed on Int-Per-Imp measured through TSI (Table 3; P = 0.03).

#### Behavioral Problem (BPr)

CPRS-R BPr score was negatively affected by M3 “T” allele (Add value = −0.05, CI −0.09 to −0.01, χ^2^ = 5.60, P = 0.02). M1 “C” documented lowering effect on the trait score (Add value = −0.07, CI −0.15 to 0.01, χ^2^ = 2.91, P = 0.08). M2 failed to exhibit any independent contribution. Haplotypes C-A (M1-M2) and A-T (M2-M3) negatively affected the trait score (Table 3) with significant confidence interval for the G-A-T haplotype (CI = −0.11 to −0.002).

#### Executive deficit

The studied variants failed to show any direct association with Barkley’s Deficit in Executive Functioning-Child & Adolescent (BDEF-CA), Short attention span (SAS) or Erratic organizational capability (EOC) (Data not presented). However, performance of probands while playing computerized games was significantly affected by different genotypes (Table 4). M1 “C” exhibited association with lower score for all the games, with large effect size (ES) for Game 2 and 4 (Cohen’s d 0.78 and 1.26 respectively). Presence of M2 “C” was associated with lower scores for Game 2 and 4 with large ES (Cohen’s d 0.88 and 0.98 respectively). No significant difference was noticed for M3 variants.

**Table 4:** Association of genotypes with scores obtained through computerized games.

Variant	Allelic combination	Game1	ES1*	Game2	ES2*	Game3	ES3*	Game4	ES4*
M1	GG	143.5 ± 72.45	0.22	70.30 ± 49.69	0.78	114.42 ± 49.69	0.19	132.48 ± 47.71	1.26
	GC	128.5 ± 65.76		40.40 ± 20.8		105.5 ± 43.13		77.45 ± 39.39	
M2	AA	146.8 ± 75.21	0.37	80.44 ± 50.05	0.88	122.39 ± 34.65	0.76	133.7 ± 41.85	0.98
	CA/CC	124.48 ± 38.68		46.55 ± 22.06		94.93 ± 39.27		97.68 ± 31.02	
M3	GG	147.86 ± 71.94	0.52	69.53 ± 51.83	0.12	130.2 ± 39.45	0.49	128.50 ± 40.83	0.47
	GT/TT	111.28 ± 69.64		74.95 ± 40.47		110.65 ± 40.79		108.41 ± 44.70	

*ES = Cohen’s D Effect size indicating standardized difference between two means.

#### Co-morbid disorders

In the studied ADHD population, 38% probands did not exhibit any co-morbidity (NONE), while 27% presented with ODD/CD, 10% had learning difficulty, 3% was with MD and 22% was under combined group exhibiting more than one co-morbid disorder. Significant association with all the three variants was detected (Table 5). M1 “GC” showed significantly higher occurrence in ODD/CD group (X^2^ = 16.5, P = 0.001, Power 96%). Significant difference was also noticed for M2 due to higher occurrence of “CC” (X^2^ = 22.8, P = 0.001, Power 99%) in the LD group (Table 5). M3 “T/TT” also showed significant difference for the LD group (X^2^ = 10.8, P = 0.01 and X^2^ = 22.8, P = 0.001 respectively, Power >90%).

**Table 5:** Association of variants with ADHD in subjects with or without co-morbidity.

Variant	Allele/Genotype	NONE	ODD/CD	LD	Combined	χ2(P)*
M1	G	0.97	0.90	0.97	0.94	6.35 (0.09)
	C	0.03	0.10	0.03	0.06	
	GG	0.94	0.79	0.95	0.88	16.5 (0.001)
	GC	0.06	0.21	0.05	0.12	
	CC	0.0	0	0	0.0	
M2	A	0.78	0.77	0.74	0.82	1.89 (0.60)
	C	0.22	0.23	0.26	0.18	
	AA	0.60	0.54	0.59	0.67	22.8 (0.001)
	AC	0.35	0.46	0.29	0.31	
	CC	0.05	0.0	0.12	0.02	
M3	G	0.65	0.68	0.47	0.61	10.8 (0.01)
	T	0.35	0.32	0.53	0.39	
	GG	0.41	0.48	0.24	0.40	21.9 (0.001)
	GT	0.49	0.40	0.47	0.42	
	TT	0.10	0.12	0.29	0.18	

### Influence of markers on symptomatic improvement

ADHD probands harboring rs2069459 “TT” showed improvement (Add value = 9.98,CI 1.16 to 18.8, χ^2^ = 5.87, p = 0.02) in disease severity, as measured through CPRS-R ADHD index after 3 years (Table 6). Effect of other variants was insignificant.

**Table 6:** Quantitative Trait analysis involving gene variants and improvement in ADHD index.

rs ID	Allele/Genotype	Add value	CI	Χ^2^ (P)*
M1	C	0.23	−5.19 to 5.65	0.01 (0.93)
	GC	0.25	−5.43 to 5.94	0.01 (0.93)
M2	C	−0.84	−4.47 to 2.80	0.21 (0.65)
	AC	−0.50	−5.11 to 4.10	0.02 (0.90)
	CC	−2.27	−11.86 to 7.32	0.19 (0.66)
M3	T	2.82	−0.09 to 5.72	3.64 (0.05)
	GT	1.42	−2.78 to 5.61	0.005 (0.94)
	TT	9.98	1.16 to 18.8	5.87 (0.02)

## Discussion

This pioneering investigation in the field of ADHD showed significant contribution of three *CDK5* variants in the phenotypic attributes. *CDK5* is located at 7q36 and the transcript contains 12 exons^28^. In the present study, three genomic regions were analyzed. Exon 2 and its flanking regions failed to show any bimorhic SNP. Two stretches including Exon 6 (Ensemble genome browser 84, location 151053818–151054597) and Exon 10 (Ensemble genome browser 84, location 151055410–151056277) revealed presence of three bimorphic variants in the studied Indian population.

rs2069454 (M1) “C” was identified as a transcriptional activator and frequency of this allele was very low in the studied population. The “C” allele, as part of haplotype also was scanty in the probands. On the other hand, a nominal transmission bias was observed for this allele from mothers below the age of 30 years as opposed to those more than 30 years of age during the birth of the child. This allele, as part of the haplotype “C-A” (M1-M2), was associated with low trait score for BPr and DSM-IV-TR-Imp. An earlier study reported association of higher maternal age (mean 33.3 ± 5.0 years) with oppositional behavior score and HA/Imp score of ADHD probands^29^. Our observation on rs2069454 “C” indicated that lower Bpr and Imp of probands born to younger mothers (<30 years) could be, at least partially, due to the *CDK5* rs2069454 “C” variant. On the other hand, the “G” allele, which may lower transcriptional efficiency, as part of haplotype G-C (M1-M2) showed association with increase in trait score for IA and haplotype G-A-T (M1-M2-M3) showed association with low trait score for Int-Per-Imp and BPr. “GG” genotype frequency was low in subjects with co-morbid ODD/CD with concomitantly higher frequency of the “GC” genotype. From these observations it could be speculated that rs2069454 (M1) has a dimorphic role in ADHD associated traits which needs further in depth analysis in large number of samples, since stratification of subjects based on co-morbid disorders lowered the number of subjects in each group.

rs2069456 (M2) “AC” heterozygous genotype frequency was significantly higher in families with ADHD probands while a parental transmission bias was noticed for the “A” allele, which was chiefly maternal in nature. Haplotypes showing higher occurrence also had the M2 “A” as their part. QTA exhibited significantly low score for Int-Per-Imp and Bpr in presence of the “A” allele. On the other hand, the “C” allele and “CC” genotype positively influenced the DSM-IV-IA score and thus may be associated with higher IA. As part of haplotypes also, the “C” allele showed association with IA. *In silico* analysis suggested that in presence of the “C” allele, Cdk5 transcription could be lowered due to the absence of binding to transcription factor HSF. The present observation thus creates room for further research to identify how a reduction in Cdk5 level impairs attention to affect the etiology of ADHD.

rs2069459 (M3) was found to be a potential splice regulator, though detailed information could be not be obtained by *in silico* analysis. Population based analysis showed higher occurrence of the G-G haplotype (M1-M3) in the probands though the difference was statistically insignificant. On the other hand, the C-G haplotype (M1-M3) exhibited statistically significant higher occurrence in the control population and thus the “G” allele could be considered as a protective factor towards ADHD. In contrast to that, frequency of the “T” allele was significantly higher in ADHD probands with co-morbid LD. As part of various haplotypes, the “T” allele showed association with low trait scores for HA, Imp, and Bpr. ADHD probands with “T” allele or “TT” genotype showed significant improvement during follow up as compared to those with “GG”. It could be hypothesized from the above data that the “T” allele may have a role in the etiology of ADHD and co-morbid LD, which merits further investigation.

Apart from its various roles in development^26^, Cdk5 regulates the exocytosis process of synaptic vesicles through phosphorylation of synapsin I, Munc18, and P/Q subtype voltage-dependent calcium channel^30^. Cdk5 negatively regulates postsynaptic signaling of striatal DA and thus could be speculated to have a role in post-synaptic receptor regulation^31^. Blocking of DA reabsorption in the axon terminal is induced by chronic cocaine addiction due to activation of delta-Fos B transcription factor, an upstream regulator of Cdk5. Moreover, elevated level of Cdk5 after chronic cocaine exposure may lead to altered signaling through DA receptors to produce adoptive behavioral response^32^. Cdk5 also modulates cognition as a part of the penumbra of function. Epigenetic modulation of *CDK5* was proposed to lead to differential gene expression in the nucleus accumbens ultimately changing stress related and drug abusive behaviors^33^. In experimental animals, differential Cdk5 expression was observed under chronic and acutely stressed conditions^34^. The present study also revealed differential role of *CDK5* variants in modulating ADHD associated traits and thus warrants further in depth analysis on the contribution of Cdk5 in the etiology of the disorder.

**Figure 2:**
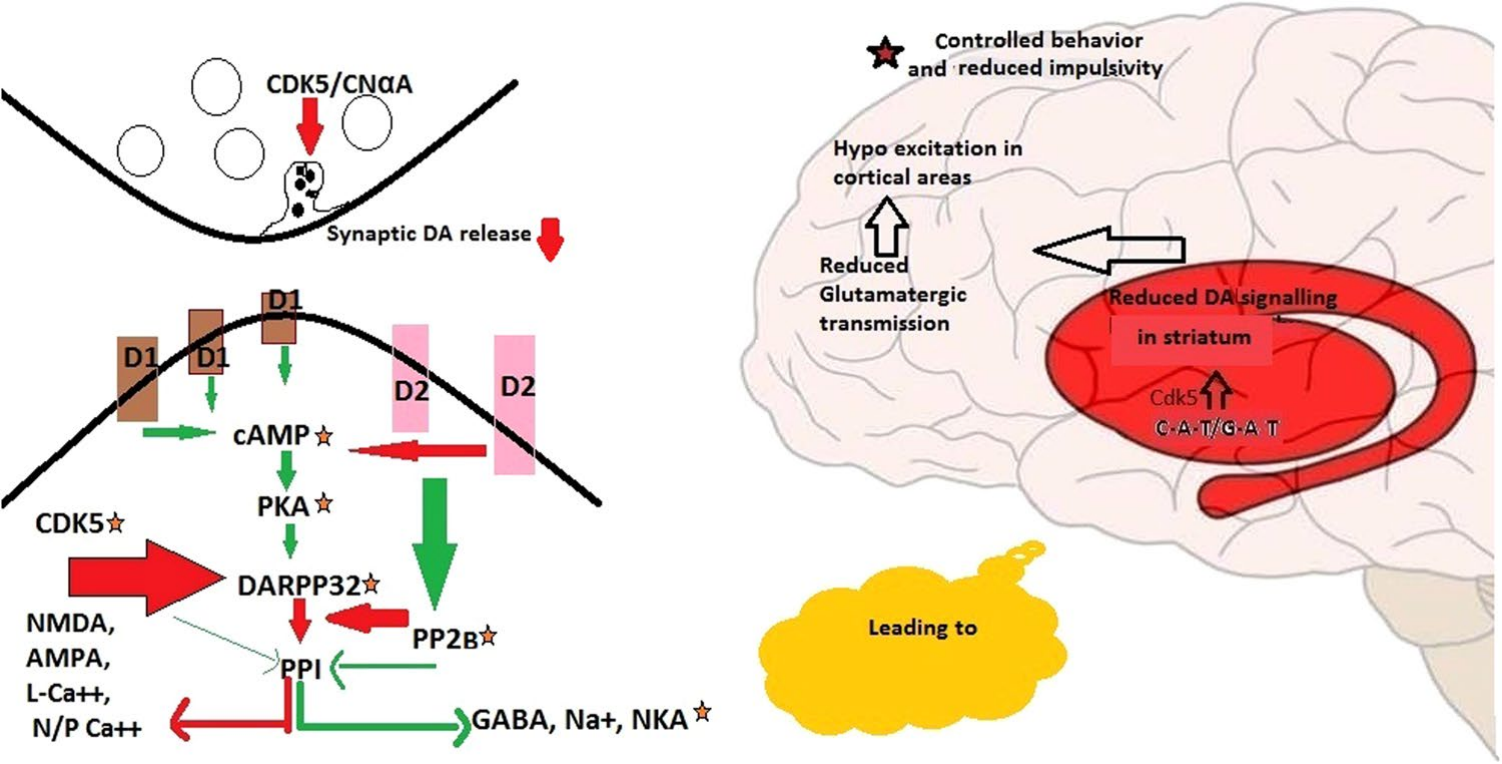
Proposed role of CDK5 haplotypes CAT and GAT in ADHD through modulation of dopaminergic transmission.  lines indicate activation and  lines indicate deactivation or suppression. Pre-synaptic exocytosis is guided by optimum balance between Cdk5 and CNαA^30^. Straital DA release in the striatum is inhibited by Cdk5^34^. Thus synaptic DA availability is low in presence of Cdk5. In the post synaptic cell, D1 like receptors activate glutamate signaling through PPI inactivation while D2 type receptor maintains base line homeostasis by PPI activation indirectly by the effect of PP2B^55^. Activated Cdk5 phosphorylates 75^th^ Threonine residue of DARPP32 resulting into its loss of activity^36^. Nonfunctional DARPP32 can no longer inhibit PP1 and resting state is maintained where NMDA, AMPA, Ca^++^ channels are inactive, while GABA & NKA are active.

We have performed *in silico* functional analysis to identify the role of the gene variants. rs2069454 (M1) “C” was identified as a transcriptional activator by influencing binding with NKX2. rs2069456 (M2) “A” allele was also predicted to act as a transcriptional activator during stress by HSF binding. Earlier study showed that Cdk5 may act as a negative regulator of post-synaptic striatal DA^31^ and presynaptic DA release^35^. Through DARPP32 phosphorylation also, Cdk5 may attenuate DA signaling^36^. Image analysis of ADHD patient’s brain showed hypo-activation of the fronto-parietal network^18^. We hypothesize that *CDK5* haplotypes C-A-T and G-A-T, which was predicted to increase transcription, may inhibit DA transmission leading to reduced cortical excitement (Fig. 2). This may eventually reduce the level of uncontrolled behavior and Imp. On the other hand, the G-C haplotype (M1-M2) may reduce Cdk5 transcription, thereby hyperstimulating dopaminergic transmission leading to lack of attention. Data obtained in the present study support the above notion since the G-C haplotype showed association with an increase in IA score.

For measuring the EF deficits in ADHD individuals, two major theories have been proposed^37^. One is to measure EF deficit through different test batteries like Stop Signal Test, Wisconsin Card Sorting Test, Tower of Hanoi, Tower of London, etc, all of which primarily deals with working memory and response inhibition. The other way is to measure EF as a function of self regulation and inhibition^38, 39^. It was proposed that the study design for such assessments should be designed in such a way that it could justify the functions over long period of time rather than short momentary laboratory based tests, since “measures taken in clinics or laboratory assessments over relatively brief temporal durations are going to prove less sensitive to the identification of the disorder and its associated cognitive deficits than will measures collected repeatedly over longer time periods”^37^. In the present study, both the aspects were taken into consideration by using computerized games as well as structured questionnaire, BDEF-CA and SAS/EOC. Though the structured questionnaires failed to exhibit any direct correlation, computer based performance analysis showed significant effect sizes for M1 and M2 “C” variants and low score for Game 2 and Game 4. While Game 2 was used for estimating Speed of information processing, Game 4 measured concentration through Dual N-back test^40^. On the basis of these findings, it can be interpreted that individuals harboring these CDK5 variants may have better cognitive function, while the opposite could be true for the other allelic variants. However, the games were played by only limited number of probands warranting replication of this study in higher number of subjects for validation.

Impaired spatial learning and memory was reported in Cre ER knockout mice (conditional deactivation of CDK5R1 making Cdk5 nonfunctional) due to disproportionate dendritic spine density in the CA region of the hippocampus^41^. Since the alleles M1 “G” and M2 “C” were predicted to reduce Cdk5 transcription, presence of the G-C haplotype may lower the level of Cdk5 hampering the process of sustained attention finally affecting long term potentiation. The role of M3 was not clear from the *in silico* analysis. The marker was in strong LD with rs2069456 in the ADHD probands. Being in LD with M2, M3 “T” allele may increase Cdk5 level in presence of M2 “A” thereby improving the trait scores in existence of C-A-T or G-A-T haplotypes.

ADHD was reported to have a male biasness^42^ and generally manifested at an early age^2^. However, quantitative trait analysis on CDK5 variants failed to exhibit any significant effect of gender and age on the trait scores.

Allelic variants of the studied markers, M1 “G”, M2 “C” and M3 “T”, as part of haplotypes showed association with reduction in HA score. On the other hand, in a previous study on CDK5R1 knockout mice, with non functional Cdk5, HA comparable to ADHD was documented^27^. Whether this difference in the level of HA is due to differences between species or is due to inclusion of only limited number of gene variants warrants further in depth analysis on CDK5 to identify its actual role.

Major limitations of the present study are (1) limited number of samples, (2) association of only few variants, and (3) lack of functional validation of the studied SNPs. However, the studied sample size met the criteria for being statistically significant^43^ and thus could be taken into account while considering relevance of the study. Further, this novel study on *CDK5* revealed significant association of three functional gene variants with ADHD, which is quite intriguing based on the localization and role of CDK5 during the developmental period. Previous investigators suggested that *CDK5* regulate vesicular transmission, post receptor DA signaling, dendritic spine formation^30, 44, 45^, etc. thus regulating neurotransmission. Our study for the first time revealed that CDK5 may affect, (1) trait scores, (2) subtypes, (3) co-morbidity, and (4) long term outcome of ADHD probands. Thus, this novel study on limited number of *CDK5* variants indicate a possible role of gene variants in the etiology of ADHD which merits further validation in a large cohort of subjects belonging to different ethnic groups and functional validation of gene variants.

## Materials & Methods

### Participants and study design

#### Inclusion criteria

Unrelated nuclear families with ADHD probands (*n* = 217; mean age 8.10 ± 3.33; sex ratio M:F 8.43:1) were recruited based on the Diagnostic and Statistical Manual for Mental Disorders-IV-text revised (DSM-IV-TR) criteria^2^. ADHD probands were assessed by the Conners’ Parent Rating Scale-Revised (CPRS-R)^46^ to measure the presence and severity of symptoms. Intelligence/developmental quotient were determined by the Wechsler’s Intelligence Scale for children^47^ for probands above five years and Developmental Screening Test for children below 5 years^48^ respectively. Out of 217 probands, 174 were complete parent-proband trios, 33 had only one parent while 10 were affected probands only. Majority of the probands belonged to the combined subtype (73.0%) while hyperactive/impulsive (12%) and inattentive (15%) subtypes were only limited.

Ethnically matched control subjects were evaluated using the DSM-IV-TR criteria for ADHD, hypothyroidism, intelligence/developmental quotient (>80) as well as for any psychiatric disorder running in the family and those without any abnormality (*n* = 250; age range: 4–20 yrs; sex ratio 0.85:1) were recruited for the study. Informed written consent was obtained for participation in the study. The study protocol was approved by the Human Ethics Committee of Manovikas Kendra (02–110414) which follows the guidelines of the Indian Council of Medical Research. All methods were performed in accordance with the relevant guidelines and regulations of the Committee.

#### Exclusion criteria

Subjects with only psychiatric problems including pervasive developmental disorders, any form of mental retardation (IQ ≤ 70) and fragile-X syndrome, were excluded.

### Assessment of traits

Inattention, hyperactivity, impulsivity were assessed through two different testing tools. For inattention and hyperactivity, traits were measured through DSM-IV-TR and CPRS where as impulsivity was measured though DSM-IV-TR^2^. Tsukayama Scale of Impulsivity (TSI)^49^ was used to measure domain specific impulsivities, like inter-personal impulsivity. Behavioral problem and disease severity were measured through CPRS^46^.

#### Analysis on age of onset

Age of onset was considered as the time when the symptoms were disruptive enough to call for a remediation. Sex and age were considered as phenotypic covariates.

#### Assessment of co-morbidity

Co-morbid conditions were assessed using the DSM-IV-TR criteria. To investigate association between a variant and co-morbidity, probands were classified into four categories; without any co-morbidity (NONE), with co-morbid behavioral problem (ODD + CD), with co-morbid learning difficulty (LD) and with more than one co-morbidity (Combined). Probands were also classified based on the most prominent phenotypes as inattentive (IAS), hyperactive/impulsive (HAS) and combined exhibiting both hyperactivity/impulsivity as well as inattentiveness (CS).

### Assessment of executive function

Barkley’s Deficit in Executive Functioning-Child & Adolescent (BDEF-CA) scale was used to assess the executive impairment in ADHD probands^50^. Additionally, structured questionnaire designed from DSM-IV-TR and CPRS was used to measure Short Attention Span (SAS) and Erratic Organizational Capability (EOC) in the probands as detailed earlier^40^. Computerized games were used to find out the effect sizes of the polymorphisms on the performance^40^. 100 probands were called for participating in the game, few could not come due to health issues, few due to personal reasons, while few were reported to leave the station. Finally only 25 probands turned out to participate in the assessment using computerized games.

#### Reassessment of traits

CPRS-R was used for reassessment of ADHD associated traits after 3 years. Improvement index was calculated as 1 − T_n_/T_0_ (T_n_ = post-treatment score, T_0_ = initial score).

### Analysis of gene variants

Online program F-SNP (compbio.cs.queensu.ca/F-SNP) was used to analyze functional roles of the selected variants. NCBI-CCDS was used to identify functional regions of CDK5. Peripheral blood leukocytes were processed for extraction of genomic DNA^51^. Oligonucleotides designed using the Primer3 program (www.bioinformatics.nl/primer3plus/) were used for PCR amplification in ABI Gene Amplifier #9700 PCR system. SNPs were analyzed by sequencing of the PCR amplicon in Applied Biosystems 3130 Genetic analyzer using Big Dye v 3.1 chemistry and Sequencing Analysis Software, v 5.2. Detailed protocol is given in supplementary material (ST2).

## Data analysis

### Association analysis

Hardy-Weinberg equilibrium (HWE) was analyzed using the online software (http://ihg.gsf.de/cgi-bin/hw/hwa1.pl-hwe). Unphased version 3.1.7^52^ was used for population- and family-based analysis. Association with haplotypes was also analyzed using Unphased^52^. All the results were obtained following 1000 permutation, which takes care of the multiple corrections. For genetic association test, power of the significant results was calculated through Piface, v 1.76^53^.

### Analysis of Linkage disequilibrium

Linkage Disequilibrium was calculated using the Haploview version 4.1^54^.

### Analysis of Odds Ratio

Odds Ratio (OR) was calculated online (http://www.hutchon.net/confidor.htm) considering data for the wild type allele in case with respect to control for population based study, and transmitted to non transmitted in family based study.

### Genotype-phenotype correlation analysis

Association of alleles or haplotypes with age of onset, ADHD associated traits, and ADHD index obtained during follow up were analyzed by quantitative trait analysis (QTA) through Unphased version 3.1.7. 1000 times permutation was used for corrections for multiple testing. To calculate the effect size correlation for the difference in mean scores obtained through computerized games and different genotypes, Cohen’s d was calculated online (http://www.uccs.edu/~lbecker/).

## Electronic supplementary material

Supplementary Dataset 5
