# Supplementary material for: Cyclin-dependent Kinase 5: Novel role of gene variants identified in ADHD
Source: Sci Rep. 2017 Jul 28;7:6828. doi: 10.1038/s41598-017-06852-2 (PMC5533779; doi:10.1038/s41598-017-06852-2)
Supplement: Supplementary file 1 — Supplementary Dataset 5 [file 41598_2017_6852_MOESM1_ESM.doc]

**Cyclin-dependent Kinase 5: Novel role of gene variants identified in ADHD.**

Subhamita Maitra, Mahasweta Chatterjee, Swagata Sinha, Kanchan Mukhopadhyay^*^.

Manovikas Biomedical Research and Diagnostic Centre, E.M. Bypass, Kolkata, India.

*Corresponding author:

Kanchan Mukhopadhyay,

Manovikas Biomedical Research and Diagnostic Centre,

482, Madudah, Plot I-24, Sec.-J, E.M. Bypass, Kolkata - 700107, India.

Tel: 91-033-4001-9179; Fax: 91-033-2442-8275;

Email: [kanchanmvk@yahoo.com](mailto:kanchanmvk@yahoo.com)

ST1. Expression of Cdk5 in different brain regions obtained from BGEE, a database for gene expression evolution available at bgee.org.

| **Region** | **Developmental Stages** | **Source** |
| --- | --- | --- |
| **Frontal cortex** | 5th month of gestation | Affymetrix, DNA micro array |
|  | 6-12 years |  |
| **DLPFC** | Infant stage (0-3 years) |  |
|  | 2-5 years |  |
|  | 6-12 years |  |
|  | Adolescent (12-20 years) |  |
| **Ammon’s horn** | 12-20 years |  |
| **Caudate Nucleus** | Infant (0-3 years) |  |
|  | 2-5 years |  |
|  | 6-12 years |  |
|  | Adolescence |  |

ST2. List of *CDK5* variants studied and their putative role. Variants presented in bold were found to be bimorphic in the studied population.

| **rs ID** | **Designated # in the tables** | **Alleles** | **Ancestral allele** | **Function** |  |
| --- | --- | --- | --- | --- | --- |
| **rs2069453** |  | C/T | C | Transcriptional activation  T: MATA 1 | Sense 5- GTGCCAGGTGCTTTTCTAGG- 3’  Antisense 5’-TAGAGGACTGGGGAGGAGGT - 3’ |
| **rs2069454** | M1 | G/C | G | Transcriptional activation  C:NKX2 |
| **rs2069455** |  | C/T | T | Transcriptional activation  C:Sp1 |
| **rs1057766** |  | C/T | C | Splicing regulation, no allelic discrimination |
| **rs2069456** | M 2 | C/A | A | Transcriptional activation  A: binding of HSF |
| **rs2069459** | M 3 | G/T | G | Essential splice site | Sense 5’-GAGGGTAAAGGGAGGGTGAG- 3’  Antisense 5’- GGGAAAGGAGCCAATTTATGA- 3’ |
| **rs11541602** |  | G/T | G | T: Splicing enhancement |
| **rs2069460** |  | C/T | C | Transcriptional activation  C:CF1 binding |

ST3. Comparison of allelic and genotypic frequencies among subtypes**.**

| **Variant** | **Allele/**  **Genotype** | **Inattentive** | **Hyperactive** | **Combined** | **χ2(P)*** |
| --- | --- | --- | --- | --- | --- |
| **M1** | G | 0.89 | 0.98 | 0.96 | 8.36  (0.02) |
|  | C | 0.11 | 0.02 | 0.04 |  |
|  | GG | 0.79 | 0.95 | 0.91 | 13.5  (0.001) |
|  | GC | 0.21 | 0.05 | 0.09 |  |
|  | CC | 0 | 0.0 | 0.0 |  |
| **M2** | A | 0.77 | 0.92 | 0.77 | 21.9  (0.0001) |
|  | C | 0.23 | 0.08 | 0.23 |  |
|  | AA | 0.58 | 0.84 | 0.57 | 22.0  (0.0001) |
|  | AC | 0.38 | 0.16 | 0.39 |  |
|  | CC | 0.04 | 0.0 | 0.04 |  |
| **M3** | G | 0.59 | 0.57 | 0.65 | 1.45  (0.49) |
|  | T | 0.41 | 0.43 | 0.35 |  |
|  | GG | 0.35 | 0.35 | 0.42 | 3.50  (0.48) |
|  | GT | 0.48 | 0.43 | 0.45 |  |
|  | TT | 0.17 | 0.22 | 0.13 |  |

ST4. Population-based comparative analysis on haplotype frequency

| **Variant** | **Haplotype** | **Control** | | **Probands** | | | | | |
| --- | --- | --- | --- | --- | --- | --- | --- | --- | --- |
|  |  | All | Male/  Female | All | | Male | | Female | |
|  |  |  |  | Freq | χ2(P)* | Freq | χ2(P)* | Freq | χ2(P)* |
| **M1-M2** | G-A | 0.70 | 0.71/0.69 | 0.72 | 0.62(0.43) | 0.72 | 0.15 (0.70) | 0.76 | 0.78(0.37) |
|  | G-C | 0.23 | 0.22/0.24 | 0.22 | 0.35(0.55) | 0.22 | 0.09 (0.77) | 0.21 | 0.20(0.66) |
|  | C-A | 0.05 | 0.04/0.07 | 0.06 | 0.001(0.97) | 0.06 | 0.48 (0.49) | 0.03 | 0.79(0.38) |
|  | C-C | 0.02 | 0.03/0.0 | 0.0 | 2.07(0.15) | 0.0 | **4.37 (0.036)** | 0.0 | 0.0(1.0) |
| **M2-M3** | A-G | 0.42 | 0.44/0.40 | 0.43 | 0.30(0.59) | 0.43 | 0.002 (0.97) | 0.47 | 0.77(0.38) |
|  | A-T | 0.34 | 0.34/0.35 | 0.35 | 4.19 ^e-005^ (0.99) | 0.35 | 0.003(0.96) | 0.34 | 0.03(0.86) |
|  | C-G | 0.20 | 0.18/0.22 | 0.20 | 0.02(0.89) | 0.20 | 0.21 (0.64) | 0.19 | 0.36(0.55) |
|  | C-T | 0.04 | 0.04/0.03 | 0.01 | **2.80(0.09)** | 0.01 | 2.07 (0.15) | 0.0 | 0.60(0.44) |
| **M1-M3** | G-G | 0.57 | 0.56/0.59 | 0.64 | 2.19(0.14) | 0.63 | 0.82 (0.37) | 0.70 | 1.69(0.19) |
|  | G-T | 0.36 | 0.37/0.34 | 0.32 | 0.68(0.41) | 0.32 | 0.25 (0.61) | 0.30 | 0.26(0.61) |
|  | C-G | 0.04 | 0.05/0.03 | 0.004 | **4.85(0.03)** | 0.005 | **4.34(0.037)** | 0.0 | 1.25(0.26) |
|  | C-T | 0.03 | 0.02/0.04 | 0.03 | 0.26(0.61) | 0.04 | 0.04 (0.84) | 0.0 | 2.13(0.14) |
| **M1-M2-M3** | G-A-G | 0.38 | 0.40/0.37 | 0.44 | 1.20(0.27) | 0.44 | 1.0 (076) | 0.47 | 1.59(0.21) |
|  | G-A-T | 0.33 | 0.35/0.31 | 0.31 | 0.06(0.81) | 0.30 | 1.22 (0.27) | 0.34 | 0.06(0.80) |
|  | G-C-G | 0.18 | 0.15/0.22 | 0.20 | 1.0(0.75) | 0.20 | 0.58 (0.45) | 0.19 | 0.37(0.54) |
|  | G-C-T | 0.03 | 0.02/0.03 | 0.01 | 1.57(0.21) | 0.01 | 1.23 (0.27) | 0.0 | 0.58(0.45) |
|  | C-A-G | 0.03 | 0.03/0.02 | 0 | 2.3(0.12) | 0.0 | 0.37 (0.54) | 0.0 | 0.90(0.34) |
|  | C-A-T | 0.02 | 0.0/0.05 | 0.04 | 0.14(0.71) | 0.04 | **5.41(0.02)** | 0.0 | 1.72(0.19) |
|  | C-C-G | 0.02 | 0.03/0.0 | 0.003 | **3.48(0.06)** | 0.01 | 32.73 (1.05e-008) | 0.0 | **--** |
|  | C-C-T | 0.01 | 0.02/0.0 | 0.0 | 11.65(0.001) | 0.0 |  | 0.0 | -- |

NB. Values marked in bold exhibited significant Odds ratio.

ST5: Haplotype transmission pattern obtained in nuclear families with ADHD probands

| **Variant** | **Haplotype** | **Parental** | | **Paternal** | | **Maternal** | | **LRS* (P)** | | |
| --- | --- | --- | --- | --- | --- | --- | --- | --- | --- | --- |
|  |  | T | NT | T | NT | T | NT | Parental | Paternal | Maternal |
| **M1-M2** | G-A | 0.80 | 0.76 | 0.76 | 0.81 | 0.85 | 0.71 | 5.15  (0.16) | 2.73  (0.43) | 6.46  (0.04) |
|  | G-C | 0.15 | 0.21 | 0.19 | 0.15 | 0.10 | 0.27 |  |  |  |
|  | C-A | 0.05 | 0.02 | 0.05 | 0.02 | 0.05 | 0.02 |  |  |  |
|  | C-C | 0 | 0.01 | 0.0 | 0.02 | 0.0 | 0.0 |  |  |  |
| **M2-M3** | A-G | 0.50 | 0.51 | 0.53 | 0.57 | 0.48 | 0.46 | 4.02  (0.26) | 1.67  (0.64) | 3.42  (0.33) |
|  | A-T | 0.36 | 0.29 | 0.33 | 0.29 | 0.39 | 0.30 |  |  |  |
|  | C-G | 0.14 | 0.18 | 0.14 | 0.12 | 0.13 | 0.22 |  |  |  |
|  | C-T | 0.0 | 0.02 | 0.0 | 0.02 | 0.0 | 0.02 |  |  |  |
| **M1-M3** | G-G | 0.65 | 0.69 | 0.70 | 0.63 | 0.60 | 0.74 | 5.95  (0.11) | 4.46  (0.22) | 5.01  (0.08) |
|  | G-T | 0.31 | 0.29 | 0.25 | 0.32 | 0.37 | 0.26 |  |  |  |
|  | C-G | 0.0 | 0.02 | 0.0 | 0.03 | 0.0 | 0.0 |  |  |  |
|  | C-T | 0.04 | 0.01 | 0.05 | 0.02 | 0.03 | 0.0 |  |  |  |
| **M1-M2-M3** | G-A-G | 0.49 | 0.50 | 0.51 | 0.54 | 0.46 | 0.46 | 5.69  (0.34) | 4.01  (0.55) | 5.40  (0.25) |
|  | G-A-T | 0.33 | 0.28 | 0.27 | 0.29 | 0.39 | 0.27 |  |  |  |
|  | G-C-G | 0.14 | 0.17 | 0.17 | 0.10 | 0.12 | 0.24 |  |  |  |
|  | G-C-T | 0 | 0.03 | 0.0 | 0.02 | 0.0 | 0.03 |  |  |  |
|  | C-A-T | 0.04 | 0.01 | 0.05 | 0.02 | 0.03 | 0.0 |  |  |  |
|  | C-C-G | 0 | 0.01 | 0.0 | 0.02 | 0.0 | 0.0 |  |  |  |

* LRS=Likelihood ratio.
